# Screening the Olive Tree Phyllosphere: Search and Find Potential Antagonists Against *Pseudomonas savastanoi* pv. *savastanoi*

**DOI:** 10.3389/fmicb.2020.02051

**Published:** 2020-08-25

**Authors:** Diogo Mina, José Alberto Pereira, Teresa Lino-Neto, Paula Baptista

**Affiliations:** ^1^Centro de Investigação de Montanha (CIMO), Instituto Politécnico de Bragança, Bragança, Portugal; ^2^BioSystems & Integrative Sciences Institute (BioISI), Plant Functional Biology Centre, University of Minho, Braga, Portugal

**Keywords:** *Olea europaea*, olive knot, biocontrol, *Bacillus amyloliquefaciens*, antagonistic mechanisms

## Abstract

Olive knot (OK) is a widespread bacterial disease, caused by *Pseudomonas savastanoi* pv. *savastanoi* (*Pss*), which currently has not effective control methods. The use of naturally occurring microbial antagonists, such as bacteria, as biocontrol agents could be a strategy to manage this disease. The objective of this work was to select bacteria from olive tree phyllosphere able to antagonize *Pss* using *in vitro* and *in planta* experiments. The elucidation of their modes of action and the potential relationship between antagonism and bacteria origin has been investigated, as well. To this end, 60 bacterial isolates obtained from the surface and inner tissues of different organs (leaves, twigs, and knots), from two olive cultivars of varying susceptibilities to OK, were screened for their *in vitro* antagonistic effect against *Pss*. A total of 27 bacterial strains were able to significantly inhibit *Pss* growth, being this effect linked to bacteria origin. Strains from OK-susceptible cultivar and colonizing the surface of plant tissues showed the strongest antagonistic potential. The antagonistic activity was potentially due to the production of volatile compounds, siderophores and lytic enzymes. *Bacillus amyloliquefaciens* P41 was the most effective antagonistic strain and their capacity to control OK disease was subsequently assayed using *in planta* experiments. This strain significantly reduces OK disease severity (43.7%), knots weight (55.4%) and population size of *Pss* (26.8%), while increasing the shoot dry weight (55.0%) and root water content (39.6%) of *Pss*-infected olive plantlets. Bacterial isolates characterized in this study, in particular *B. amyloliquefaciens* P41, may be considered as promising biocontrol candidates for controlling OK disease.

## Introduction

Olive knot (OK) disease, caused by *Pseudomonas savastanoi* pv. *savastanoi* (*Pss*), is a serious threat to olive production worldwide, especially in Mediterranean countries (Quesada et al., 2012). This disease is characterized by the formation of overgrowths (tumorous galls or knots), mainly on the olive tree branches and twigs (Quesada et al., 2010, 2012). These galls promote the decline and death of branches, leading to serious losses in terms of yield and olive oil quality (Quesada et al., 2012). Control of OK disease is difficult, being mainly based on the removal of infected branches by pruning and application of foliar sprays with copper-based compounds (Quesada et al., 2012). With such limited available options for OK disease control, the use of biocontrol agents represents a promising environmentally friendly strategy for this disease management. Indeed, several bacteria, including *Pseudomonas* (Zadeh et al., 2008; Krid et al., 2010), *Bacillus* (Krid et al., 2010, 2012) and *Rhizobium* (Kacem et al., 2009), have already displayed antagonistic activity against *Pss* under *in vitro* conditions. This antimicrobial activity was attributed to the production of bacteriocins by *Rhizobium* (Kacem et al., 2009) and *Pseudomonas* (Lavermicocca et al., 2002). However, other compounds produced by these three bacterial genera might also be involved in the inhibition of *Pss*, as previously reported for other phytopathogens (e.g., Sasirekha and Srividya, 2016; Zengerer et al., 2018). These include siderophores (Sasirekha and Srividya, 2016), lytic enzymes, antibiotics, hydrogen cyanide (Weller, 2007; Gerami et al., 2013; Zengerer et al., 2018), lipopeptides (Touré et al., 2004), and antimicrobial volatile compounds (Hernández-León et al., 2015). Nevertheless, using *in planta* assays, *Pseudomonas* was not able to suppress OK disease development (Maldonado-González et al., 2013) and *Bacillus* strains revealed a variable efficiency in reducing knot weights (Krid et al., 2012; Ghanney et al., 2016). Thus, for a most successful identification of biocontrol agents, the performance of both *in vitro* and *in planta* experiments has been recommended (De Silva et al., 2019). Ideally, such a screening process should include microorganisms, which are already adapted to the crop, as well as resident microbiota in the same environment where the biocontrol approach will be used (Ozaktan et al., 2012). This is of particular importance when considering olive tree phyllosphere-associated bacterial communities, as most of their members [living either in the surface (as epiphytes) or in the interior of plant tissues (as endophytes)] are unique to their host genotype and/or plant organ (Mina et al., 2020a). Indeed, we have previously reported a own phyllospheric bacterial community in two olive genotypes with different degrees of susceptibility to OK disease (cv. *Cobrançosa* and cv. *Verdeal Transmontana*, being the former less susceptible to OK; Mina et al., 2020a). Olive tree leaves and twigs from each cultivar also displayed different bacterial communities (Mina et al., 2020a). As far as we know, no studies have examined if the antagonistic effects of a specific bacterial strain is related to the origin of the isolate, in terms of host cultivar and/or plant organ.

In the present study, the antagonistic activity of epiphytic and endophytic bacteria, isolated from leaves, twigs and knots of two olive cultivars displaying variable susceptibilities to OK (cvs. *Cobrançosa* and *Verdeal Transmontana*), was evaluated against *Pss* through *in vitro* assays. Their antagonistic mode of action was investigated by the production of lytic enzymes, siderophores, and antibacterial volatile compounds. The ability of the most antagonistic isolates to control OK disease was further evaluated by performing *in planta* assays (olive pot experiments). This study aims to answer the following questions: (i) Is the antagonistic effect displayed by bacteria against *Pss* linked to their origin in terms of host (i.e., genotype susceptibility to OK), plant organ (i.e., leaf, twig, knot) and/or microbial habitat (epiphyte vs. endophyte)? (ii) Which mechanisms are involved in the antagonistic effect displayed by native bacteria against *Pss*? (iii) What is the potential of native bacteria in controlling OK disease development and in reducing *Pss* population on olive phyllosphere? By combining the mechanisms of antagonistic bacterial agents with host plant features (susceptibility, type of tissue, microbial habitat), we expect to increase the likelihood of finding more effective biocontrol agents.

## Materials and Methods

### Bacterial Isolates and Inocula Production

The epiphytic and endophytic bacterial isolates tested for their antagonistic effect against *Pss* were obtained from the microbial collection of the Mountain Research Center (CIMO), Instituto Politécnico de Bragança (Portugal). These isolates were originally isolated and identified from symptomless olive tree leaves and twigs, as well as from knots, of cvs. *Cobrançosa* and *Verdeal Transmontana* growing in Mirandela (Northeast of Portugal), as referred in Mina et al. (2020a,b). Briefly, for the isolation of epiphytes, bacterial suspensions made from pieces of plant tissues in peptone water were poured over nutrient agar plates. Endophytes were isolated from the same plant pieces, by inoculating surface sterilized plant fragments previously dissected into small segments (ca. 4–5 mm) on nutrient agar plates (Mina et al., 2020a,b). A total of 60 isolates [stored in 30% (v/v) glycerol at −80°C] were selected for this study, including five isolates from each population (2 plant cultivars × 3 plant organs × 2 microbial habitats; Supplementary Table S1). These isolates were selected based on their exclusive occurrence in a specific microbial habitat (epiphyte or endophyte), organ (leaf, twig or knot), cultivar (*Cobrançosa* or *Verdeal Transmontana*), and capacity to grow on artificial media. *Pss* strain EnVN39 was obtained from the same bacterial collection, being isolated from the inner tissues of active knots of naturally infected olive trees cv. *Verdeal Transmontana* (Mirandela, Portugal). This *Pss* isolate had been previously identified by sequencing a portion of the *ptz* gene by using specific primers (*Pss1*, 5′-TGGGTTGCTACTTGTACCGGA-3′ and *Pss2*, 5′-CCGTGTACTACGTTCAGCGAG-3; Basim and Ersoy, 2001). Bacterial inocula to be used for *in vitro* and *in planta* assays were prepared by transferring bacterial cells onto Luria Bertani agar (LBA) medium (10 g/L peptone, 5 g/L yeast extract, 5 g/L sodium chloride, 10 g/L agar). Bacteria (2-days-old cultures) were suspended on 5 mL liquid LB medium and shaken on a rotary shaker (100 rpm) for 24 or 48 h at room temperature. Bacterial cell densities were adjusted to an optical density at 600 nm (OD_600_ = 0.5), corresponding to a concentration of 10^8^ CFU/mL (as determined by plotting standard curves using *Bacillus* and *Pseudomonas* growth as standards).

### *In vitro* Antagonistic Activity

The antagonistic activity against *Pss* of bacterial isolates was assessed by dual culture assays. Two sterile filter paper discs (5 mm diameter) were placed 3 cm apart onto the surface of LBA medium (10 mL/9 cm-Petri dishes). Each disc was then soaked with 5 μL of *Pss* or antagonist and left to dry in laminar flow cabinet, before being incubated at 25 ± 2°C in the dark. Control plates were performed with single inoculated discs (for each *Pss* and antagonist). For each bacterial isolate-*Pss* combination, five replicates were done and the whole experiment was repeated twice. For each testing bacteria, daily measurements of the internal radius (i.e., the radial growth toward the interacting bacterial colony) were performed. Measurements were performed until no further growth was observed, at least for one of the interacting species. The growth rate (mm/day) of each bacterial isolate was estimated from the slope of the linear regression of the increase of radial growth of the colony (mm), along the cultivation time (in days). The percentage of growth rate reduction for both pathogen and antagonist was estimate in comparison with control plates, by using the following equation: [(*GC*-*GDC*)/*GC*]^∗^100, where *GC* is the growth rate of the *Pss*/antagonist colony in control plates and *GDC* is the growth rate of the *Pss*/antagonist colony in the dual-culture assay.

### Mechanisms Associated With the Antagonistic Activity

Bacterial strains promoting more than 50% of *Pss* growth inhibition, while not being significantly inhibited by *Pss*, were considered as displaying relevant antagonistic activity (Supplementary Table S2). These isolates were further screened for the production of different compounds related to phytopathogen biocontrol, including antibacterial volatile compounds, siderophores, and lytic enzymes (lipases and proteases).

#### Volatiles Production

A volatile assay was designed and performed for evaluating the potential effect of volatiles produced by antagonists on *Pss* growth. This assay was performed as previously described for *in vitro* antagonistic activity (see section “*In vitro* Antagonistic Activity”), but using significant modifications. Before inoculation, a long strip of agar (1 cm wide) was removed from the mid portion of LBA medium (10 mL/9 cm-Petri dishes). In one side of this plate, one sterile filter paper disc (5 mm diameter) soaked with 5 μL of *Pss* was placed in the center, whereas in the opposite side a bacterial antagonist suspension (5 μL) was spread over the agar using a sterile cotton swab. The use of LB medium instead of antagonist suspension was used as control. The plates were sealed and following incubation at 25 ± 2°C in the dark, during the same period as used in dual-culture assay, the *Pss* colony area was measured and compared with control. The percentage of growth rate reduction was determined using the formula presented in the above section. This assay was performed considering five replicates for each antagonistic-*Pss* combination and repeated twice.

#### Siderophores and Lytic Enzymes Production

Siderophores production was evaluated using Chrome azurol S (CAS) medium, prepared according to Pérez-Miranda et al. (2007). CAS medium (10 mL) was plated in 9 cm Petri dishes, followed by the application of a LBA medium overlay (10 mL). Dual culture assays were established as described above, but using CAS plates. After an incubation period at 25 ± 2°C in the dark, during the same period as used in dual-culture assay, the orange zone formed around the bacterial colonies (an indication of siderophore production) was measured.

The production of proteases and lipases was assessed through dual-culture assays, but using LBA medium supplemented with corresponding enzyme substrates, according to Maria et al. (2005). Briefly, for protease activity, dual cultures were established on LBA medium supplemented with 0.4% (w/v) gelatin (Prolabo), at pH 6.0. After an incubation at 25 ± 2°C in the dark, during the same period as used in dual-culture assay, the plates were flooded with saturated aqueous ammonium sulfate (Prolabo). As the undigested gelatin precipitates with ammonium sulfate, the appearance of a clear area around the colony is an indication of protease activity. For lipase activity, dual cultures were established on LBA medium supplemented with 1% (v/v) Tween 20 (Aldrich). A clear zone around the colony indicates the production of lipase.

For both siderophores and lytic enzymes assays were performed five replicates for each antagonistic-*Pss* combination and the whole experiment was repeated twice. Controls were performed as previously described by using single inoculated discs in each plate. The level of siderophore production and enzyme activity was evaluated by using the formula *D*-*d*, where *D* is the area of colony plus orange (for siderophores) or clearing (for enzymes) zone, and *d* is the area of colony (both in mm^2^). For each antagonist, the percentage of increase on siderophore/lytic enzyme production in the presence of the pathogen was determined by using the equation [(*ADC*-*AC*)/*AC*]^∗^100, where *AC* is the siderophore/lytic enzyme production in control plates and *ADC* is the same in dual-culture assay.

### *In planta* Biocontrol Assays

The bacterial strain that exhibited the greatest inhibitory effect on *Pss* growth in dual-culture assays (isolate P41 – *Bacillus amyloliquefaciens*) was selected for *in planta* assays (pot experiments). This study aims to assess the biocontrol ability of this isolate *in planta* against OK disease development, without compromising plant growth. As direct activation of plant defense is commonly associated with a reduced plant growth (Stenberg, 2017), the impact of isolate P41 on plant growth would be studied as well.

#### Production of Olive Plantlets

Pot experiments were performed with 2-year-old olive plantlets (cv. *Cobrançosa*), obtained from propagation of olive tree semi-wood cuttings. To improve rooting, the base of cuttings was treated with indole-3-butyric acid (IBA, 3 000 ppm) and transferred to basal heated benches filled with sand and perlite mixture (1:1). Cuttings were automatically sprayed for 10 s, every 40 min, and kept under greenhouse conditions (day/night thermal regime of 23°/18° ± 2°C, 10 h light/14 h dark photoperiod and 70 ± 10% relative humidity) for 3 months. Rooted cuttings were then selected and transplanted to plastic pots of two liters, containing the same growth mixture as before, and maintained for 2 years under the same greenhouse conditions. During this plant growth period, plants were irrigated every 2 days.

#### Plant Inoculation

Both *Pss* and antagonistic isolate (P41) inocula were prepared as previously described in section “Bacterial Isolates and Inocula Production,” but using LB media containing 1% (w/v) of agar. Inoculation of olive plantlets with *Pss* and/or antagonistic P41 isolate was performed according to Penyalver et al. (2006) with minor changes. A V-shaped wound (1 cm long, 5 mm wide, and about 2 mm deep) was made on the main stem with a sterile scalpel. This wound was used for performing the plant inoculations with 10 μL of *Pss* and/or selected antagonist (P41). Controls were inoculated with 10 μL sterile LB culture medium containing 1% (w/v) of agar. Following inoculation, each wound was wrapped with Parafilm, which was removed 1 week later. For each treatment (*Pss*, P41, and *Pss* + P41) and control, a total of 30 olive plantlets were inoculated, in a total of 120 plants. All plants were maintained at 26°C, 8 h light/16 h dark photoperiod and 70% of relative humidity, and watered when needed.

#### Evaluated Parameters

Disease rating was visually recorded after 14, 28, 42, 56, and 70 days post-inoculation (DPI) by using the severity scale of Matas et al. (2012), where 1 = no knots; 2 = mild wound thickening; 3 = small knot at the wound base; 4 = small knots at both the base and top of the wound; 5 = knot completely covering the wound; 6 = knot larger than the wound (Supplementary Figure S1). OK disease development was monitored in the same 10 arbitrarily selected trees of each treatment throughout the assay. The obtained data were used to calculate the area under the disease progress curve (AUDPC) for each treatment using the following formula (Madden et al., 2007):

$$\mathrm{AUDPC}=\sum_{i=1}^{\text{n}-1}\left[\left(Y_{i}+Y_{i+1}\right)\,/\,2\right]\,\left(t_{i+1}-t_{i}\right)$$

where *Y*_*i*_ represents disease severity (1 to 6 scale) on the *i*th date, *t*_*i*_ is the time in days at the ith observation, and *n* is the total number of observations used to record OK disease development. For each time point (14, 28, 42, and 56 DPI), five plants were randomly selected and brought to the laboratory, for estimation of knot weight *per* plant, assessment of *Pss* population sizes, and evaluation of plant growth. At 70 DPI, the monitored plants used for disease severity were also used for these evaluations. The collected plants were firstly separated into leaves, stems and roots. The average knot fresh weight *per* plant was estimated by weighting the stem fragments comprising the 1 cm long wound used for the inoculation. The obtained fragments were further used to estimate *Pss* population densities. For this, the stem fragment was cut in small pieces, which were immersed in 5 mL of peptone water (10 g/L peptone, 5 g/L sodium chloride) and shaken for 10 min at 100 rpm at room temperature. Suspension aliquots (1 mL) were plated in triplicate in 10 mL of PVF-1 medium (10 mL/L glycerol, 30 g/L sucrose, 2.5 g/L Difco casamino acids, 1.96 g/L K_2_HPO_4_.3H_2_O, 0.4 g/L MgSO_4_.7H_2_O, 0.4 g/L SDS, 16 g/L agar, pH adjusted to 7.1 with HCl) and incubated at 25 ± 2°C, in the dark, until bacterial growth. Daily observations were performed in order to count the fluorescent bacteria *Pss* colonies. Results are presented as CFU *per* mL. Plant development was evaluated by registering total shoot height and root length. Shoot height was registered on the beginning of the assay and in each recording date (14, 28, 42, 56, and 70 DPI). Harvested plants were used to evaluate roots length, as well as stems and roots fresh (fw) and dry (dw) weights. For this, stems and roots were separately used to determine fresh weight (fw), oven-dried at 60°C for 3 days, and then weighed again to determine dry weight (dw). Water content was determined by dry weight/fresh weigh ratio and expressed as a percentage. Leaves near the inoculation site were used for measuring the photosynthetic pigment contents. Chlorophyll *a* (chl *a*), chlorophyll *b* (chl *b*), and carotenoids (car) contents were determined spectrophotometrically after methanolic extraction of fresh leaves, according to Ozerol and Titus (1965). Total chlorophyll was calculated by the sum of both chlorophyll *a* and *b* content. Results are presented as mg of pigment *per* g of leaf.

### Statistical Analysis

Statistical analyses were carried out using R software (R Core Team, 2018). To evaluate how microbial habitat (epiphyte/endophyte), plant organ (leaves/stems/knots), and plant cultivar (*Cobrançosa*/*Verdeal Transmontana*) are related with antagonistic potential against *Pss*, a multiple factor analysis (MFA) was performed by using the *FactoMineR* package (Le et al., 2008) from R software. Data from *in vitro* and *in planta* assays were analyzed by multifactorial analysis of variance (ANOVA) and means were compared using Tukey *post hoc* test at *p*-value < 0.05 by using *agricolae* package (functions *aov* and TukeyHSD, respectively) from R software.

## Results

In this work, the bacterial isolates previously obtained from the phyllosphere (epiphytes and endophytes) of two olive cultivars (*Cobrançosa* and *Verdeal Transmontana*, more tolerant and susceptible to OK disease, respectively) and plant organs (leaves, twigs and knots) (Mina et al., 2020a,b) were further studied (Supplementary Table S1). The 60 isolates belonged to 22 different bacterial genera and to 40 species.

### Bacteria Colonizing Different Cultivars and Microbial Habitats Displayed Different Inhibitory Effects Against *Pss*

**FIGURE 1:**
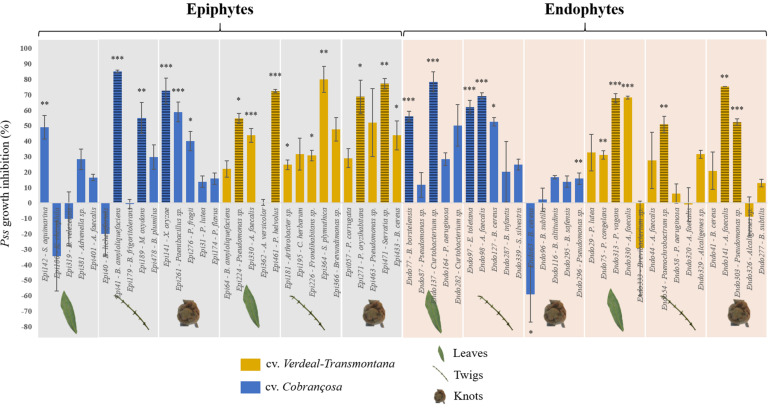
Growth inhibition of *Pseudomonas savastanoi* pv. *savastanoi* (*Pss*) after interacting with different epiphytic and endophytic bacterial isolates, obtained from leaves, twigs and knots of olive tree cvs. *Cobrançosa* and *Verdeal Transmontana*. Results are presented as the percentage of inhibition in dual-culture assays considering single inoculated controls. Horizontal lines indicate the bacterial species further used for evaluating the inhibition mechanisms. Significant differences to control (*Pss* single culture) are indicated by asterisks (*p < 0.05; **p < 0.01; ***p < 0.001).

Almost 50% (27) of the tested isolates (15 epiphytes and 12 endophytes) were able to significantly inhibit the *Pss* growth, with inhibitions ranging from 15.8 to 85.2% (Figure 1 and Supplementary Table S2). From these, most belonged to *Pseudomonas* (8), *Bacillus* (4), and *Alcaligenes* (4) genera, representing together 59.3% of the antagonistic isolates. Interestingly, within isolates of the same species a variable antagonistic activity was observed. For example, different *B. amyloliquefaciens* and *Alcaligenes faecalis* isolates displayed contrasting antagonistic potential (Figure 1). The number of bacterial strains with capacity to significantly inhibit *Pss* growth was similar regardless of the plant organ from which they were isolated (9 isolates/organ). By contrast, differences were found among cultivars, displaying cv. *Verdeal Transmontana* a higher number of antagonistic bacteria than cv. *Cobrançosa* (15 vs. 12 isolates, respectively).

**FIGURE 2:**
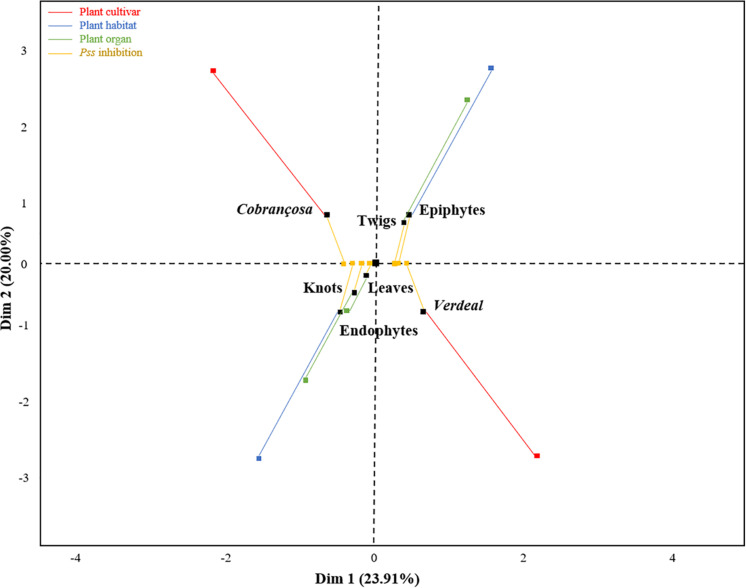
Association of *Pss* growth inhibition ability of tested bacterial isolates with plant cultivars (*Cobrançosa* and *Verdeal Transmontana*), organs (leaves, twigs, and knots) and microbial habitats (epiphytes and endophytes), as revealed by a multiple factor analysis (MFA). The first two dimensions represent 43.91% of the total variance. Dimension 1 is associated to the inhibition ability of antagonists (contribution of 77.3%, *p* < 0.001), while dimension 2 is associated to microbial habitat (contribution of 42.7%, *p* < 0.001), cultivar (41.4%, *p* < 0.001), and organ (15.9%, *p* < 0.001). An individual (black square) is at the barycenter of corresponding partial points (colored squares), thus representing an individual as seen by a single group. Therefore, each black square represents an individual viewed by two groups of variables: *Pss* inhibition (yellow squares) and their presence regarding cultivar (red squares)/microbial habitat (blue squares)/plant organ (green squares).

Using a multifactorial correspondence analysis, the inhibition of *Pss* growth by the antagonists were significantly high correlated with dimension 1 (*R*^2^ = 0.77, *p* < 0.001), while the qualitative variables (cultivar, microbial habitat and organ) were associated to dimension 2 (Figure 2). The potential to inhibit *Pss* growth was more associated to those isolates obtained from different plant cultivars (*R*^2^ = 0.59, *p* < 0.001) and microbial habitats (*R*^2^ = 0.42, *p* < 0.001), than from distinct plant organs (*R*^2^ = 0.26, *p* = 0.007). Indeed, the bacterial isolates from cv. *Verdeal Transmontana* revealed more potential to inhibit *Pss* growth (estimated coefficient = 0.64, *p* < 0.001) than those isolated from cv. *Cobrançosa*. Also, epiphytes presented higher antagonistic potential (estimated coefficient = 0.46, *p* < 0.001) than endophytes. Considering plant organs, twigs were related to isolates with higher antagonistic ability (estimated coefficient = 0.55, *p* = 0.002) than leaves or knots.

### Inhibition Mechanisms Used to Restrain *Pss* Growth

To clarify the inhibition mechanism behind each antagonistic activity, the production of antibacterial volatile compounds, siderophores and lytic enzymes (lipases and proteases) were studied when bacterial isolates were growing in co-culture with *Pss*. This study was performed only for those bacterial strains that inhibited more than 50% of *Pss* growth and were not significantly inhibited by *Pss* (Supplementary Table S2). In total, 15 antagonistic isolates (7 epiphytes and 8 endophytes), belonging to 12 genera (*Pseudomonas*, *Pseudoclavibacter*, *Serratia*, *Bacillus*, *Microbacterium*, *Xanthomonas*, *Pantoea*, *Paenochrobactrum*, *Alcaligenes*, *Brevibacillus*, *Curtobacterium*, and *Erwinia*) were selected based on these criteria.

**FIGURE 3:**
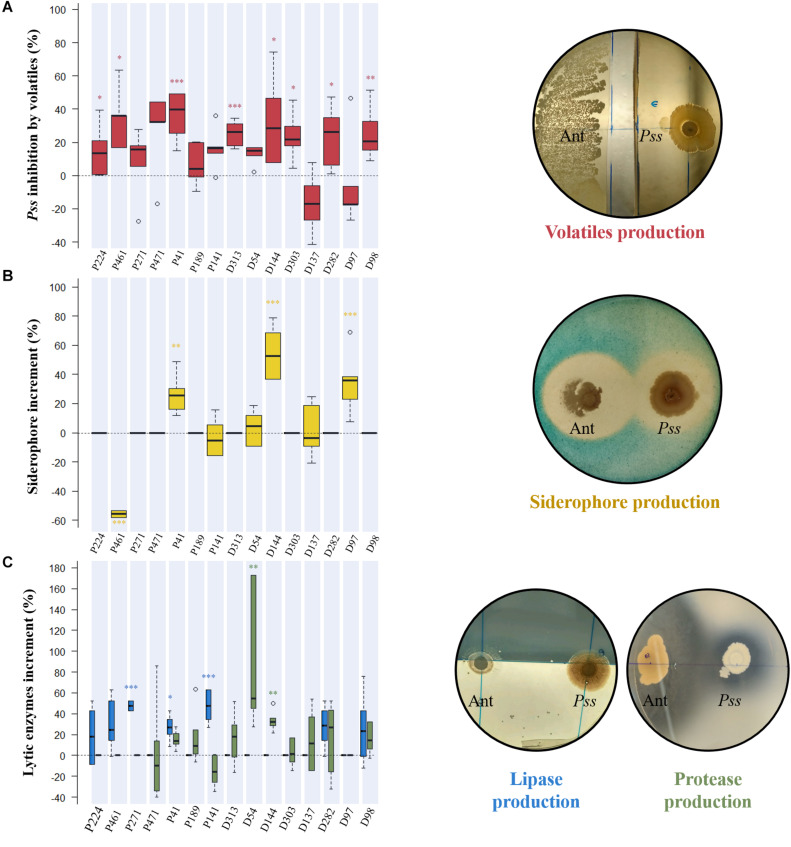
Evaluation of the inhibition mechanism displayed by each antagonistic isolate against *Pseudomonas savastanoi* pv. *savastanoi* (*Pss*). The production of antibacterial volatile compounds by antagonists was assessed by estimating their inhibitory effect on *Pss* growth (%) in dual-culture assays **(A)**, while the production of siderophores **(B)** and lytic enzymes (lipase-blue, and protease-green) **(C)** were evaluated by their increase in dual-culture assays when compared to single inoculated controls. Significant differences from controls are indicated by asterisks (*p < 0.05; **p < 0.01; ***p < 0.001). Representative plates of different dual-cultures assays with antagonist (Ant) and pathogen (*Pss*) are presented.

From all tested isolates, eight were able to significantly affect the growth of *Pss* through volatiles emission, being P41, P461, and D144 those displaying the highest inhibition rates (36.5 ± 5.8, 34.2 ± 7.0, and 32.3 ± 10.41%, respectively) (Figure 3A). The production of siderophores by bacterial antagonists was variable according to the co-culture, being significantly increased [D144 (54.4 ± 8.0%), D97 (35.1 ± 8.3%) and P41 (26.4 ± 5.4%)] or decreased [P461 (−55.8 ± 0.81%)] when compared to single inoculated controls (Figure 3B and Supplementary Table S3). When compared to control, only three epiphytic isolates significantly increased the production of lipase when challenged by *Pss* pathogen, P271 (47.5 ± 2.1%), P141 (47.1 ± 6.1%), and P41 (26.6 ± 4.8%) (Figure 3C and Supplementary Table S3). A significant increase in protease production when in co-culture with *Pss* was only observed for two endophytic isolates, D54 (87.9 ± 27.2%) and D144 (33.3 ± 4.0%).

### P41 Significantly Affected OK Disease Development, While Increasing Plant Growth

**FIGURE 4:**
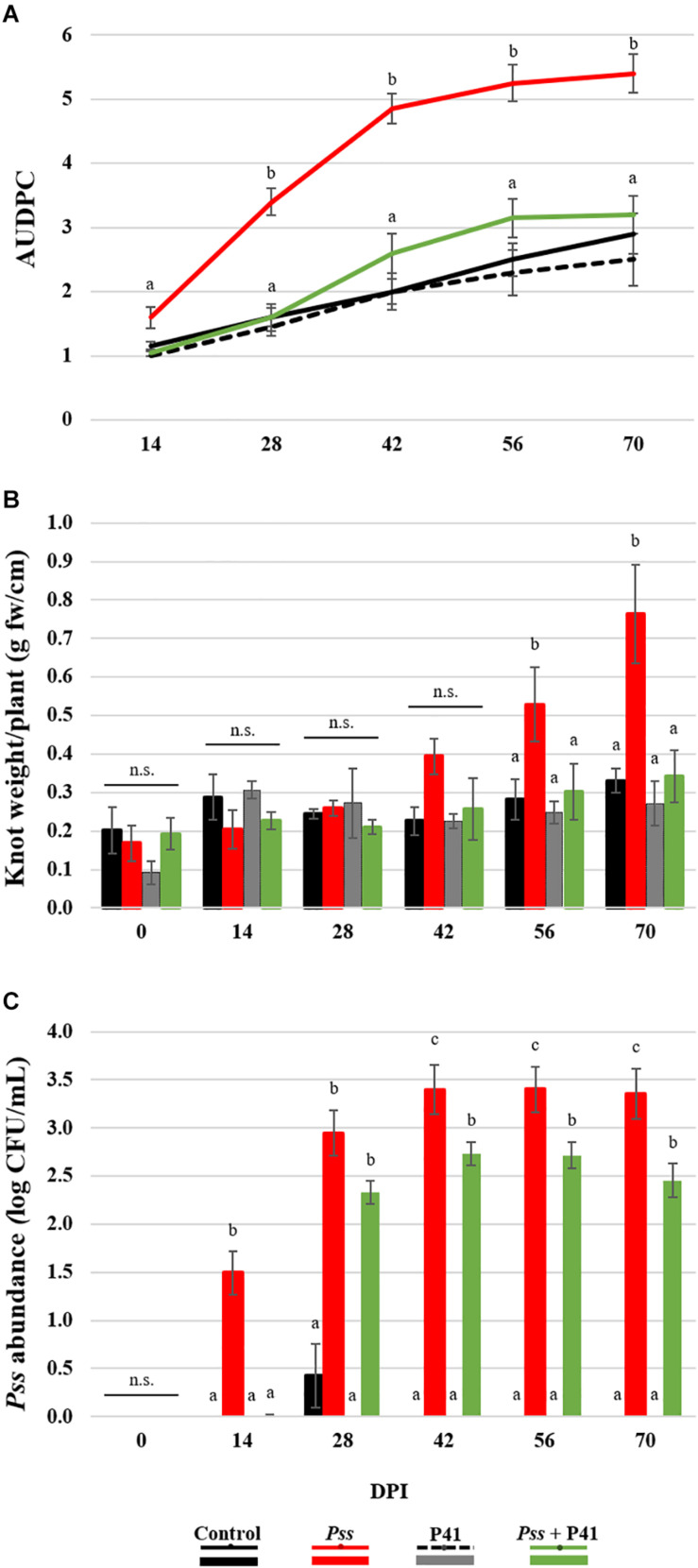
Effect of *Bacillus amyloliquefaciens* (P41 isolate) on olive knot disease progression determined by *in planta* assays. The ability to suppress disease development was evaluated by OK disease severity curves (AUDPC) **(A)**, knot fresh weight *per* plant **(B)** and *Pseudomonas savastanoi* pv. *savastanoi* (*Pss*) population density on the inoculated area of olive plants **(C)**, determined on olive plantlets inoculated with the pathogen (*Pss*), with both antagonist and pathogen (*Pss* + P41), with the antagonist (P41) and with LB medium (negative control). Data is presented as means ± SE (*n* = 10 for severity; *n* = 5 for the knots weight and *Pss* density). Statistically significant (*p* < 0.05) differences between the four treatments, in each day post-inoculation (DPI), are indicated by different letters (n.s.-non-significant).

An initial screening revealed that *B. amyloliquefaciens* (P41 isolate) exhibited the greatest inhibitory effect against *Pss* and was the greatest producer of inhibitory compounds. These features make this strain a good candidate to be explored as a biological control agent against *Pss*. Therefore, this isolate was selected for proceeding with *in planta* assays and determine its capacity to suppress OK disease development. This effect was evaluated by the progression of OK disease severity curve (AUDPC), knots weight and pathogen abundance on the inoculated area of olive plants treated with *Pss* (*Pss* alone or *Pss* + P41, Figure 4). Non-inoculated plants and inoculated with P41 were used as controls. AUDPC disease severity curve was significantly (*p* < 0.01) higher in olive plantlets solely inoculated with *Pss*, when compared to plantlets inoculated with *Pss* + P41 and controls (Figure 4A). This result was observed after only 14 DPI, being further enhanced up to the end of the assay (an overall increase of 1.8-fold, *p* < 0.01). After 56 DPI, those plantlets inoculated exclusively with *Pss* also revealed a higher knot weight (up to 2.2-fold, *p* < 0.05) when compared to plants inoculated with *Pss* + P41 (Figure 4B). In addition, the *Pss* abundance on the inoculated area become higher in *Pss* inoculated plantlets in comparison with *Pss* + P41 plantlets at 14 DPI (up to 1.3-fold, *p* < 0.05) (Figure 4C). These results were observed until the end of the assay. Despite the capacity of P41 in reducing OK disease development, some control plants (exclusively treated with P41) also developed symptoms similar to OK (Figure 4A). However, the disease severity curve did not significantly differ from non-inoculated plantlets (negative control).

**FIGURE 5:**
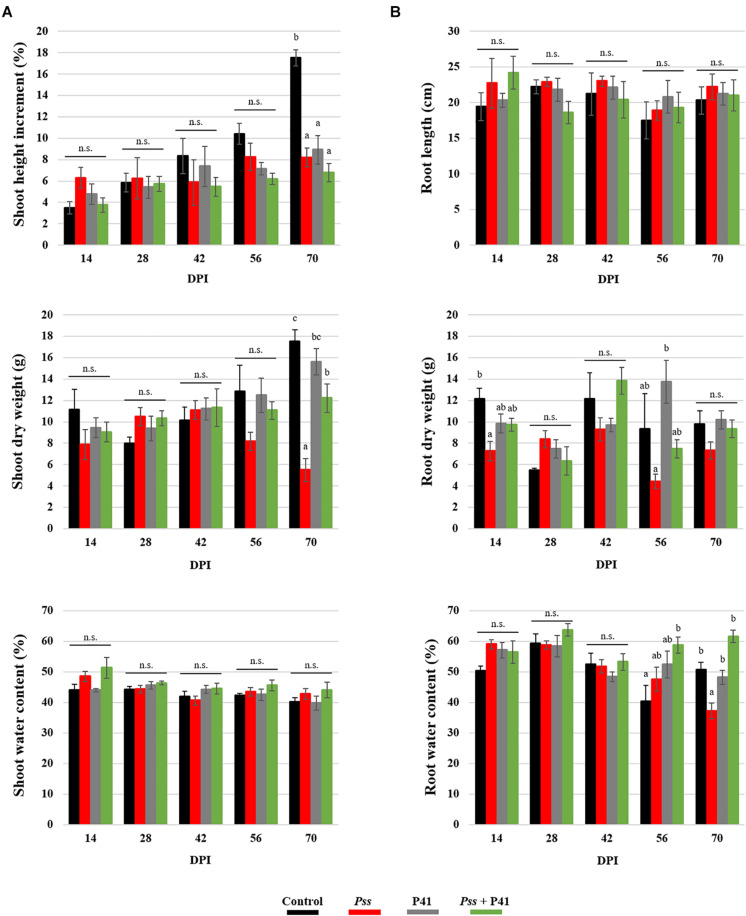
Effect of *Bacillus amyloliquefaciens* (P41 isolate) on olive plant growth determined by *in planta* assays. Plant growth parameters were evaluated at the level of shoots **(A)** and roots **(B)** of olive plantlets inoculated with the pathogen (*Pss*), with both antagonist and pathogen (*Pss* + P41), with the antagonist (P41) and with LB medium (negative control). Data is presented as means ± SE (*n* = 5). Statistically significant (*p* < 0.05) differences between the four treatments, in each day post-inoculation (DPI), are indicated by different letters (n.s.-non-significant).

The biocontrol effect of microbial agents against phytopathogens has been suggested to potentially compromise plant growth (Huot et al., 2014). Therefore, several plant growth parameters, including shoot and root length, fresh and dry weight, as well as water content and leaf pigments content, were evaluated during pot assays (Figure 5 and Supplementary Table S4). There were no significant differences on the growth parameters evaluated between plantlets inoculated with P41 and with *Pss* + P41. By the end of the assay, both displayed higher shoot dry weight (up to 2.8-fold and 2.2-fold, respectively, *p* < 0.01) and root water content (up to 1.3-fold and 1.7-fold, respectively, *p* < 0.01), when compared to plants inoculated solely with *Pss*. Inoculation of plants with any of the tested bacterial strains caused a reduction in shoot height when compared to non-inoculated plants, reaching statistically significant differences after 70 days post-inoculation. No significant differences were observed on pigment contents between treatments (Supplementary Table S4).

## Discussion

In this study, we tested the biocontrol potential of several bacteria isolated from the olive tree phyllosphere against *Pss*, by combining *in vitro* and *in planta* assays. Amongst the 60 isolates tested, it was observed, for the first time, differences in their effectiveness in inhibiting *in vitro* growth of *Pss* depending on their origin. Bacterial strains isolated from OK-susceptible cultivar cv. *Verdeal Transmontana* displayed higher antagonistic effect against *Pss* than the ones isolated from OK-tolerant cv. *Cobrançosa*. This apparent contradiction led us to hypothesize that isolates from both olive cultivars may use different modes of action to protect host plant from *Pss* infection. Microbial antagonists may use a battery of mechanisms against phytopathogens, broadly classified into direct or indirect, depending respectively on the requirement of interspecies physical contact or not (Hajek and Eilenberg, 2018). Isolates from cv. *Cobrançosa* could stimulate/prime the immunity of host plant to indirectly combat *Pss* invasions, while isolates from cv. *Verdeal Transmontana* seem to act directly against the pathogen. However, this assumption still needs to be confirmed with further work. We also found that epiphytes exhibit a higher inhibition potential against *Pss* than endophytes, which makes epiphytes as the first layer of plant defense, comprising the most promising agents for OK disease biocontrol. This aspect is of particular importance for this pathosystem, since the infection of olive tree is believed to be caused by the epiphytic *Pss* (Quesada et al., 2010). From the 27 isolates that significantly inhibited *Pss*, the antagonistic effect was highest within *Pseudomonas*, *Bacillus*, and *Alcaligenes*. Both *Pseudomonas* and *Bacillus* have been reported to be the most promising biocontrol agents of several plant diseases, in particular the ones affecting roots (Chowdhury et al., 2015; De Vrieze et al., 2018; Fan et al., 2018). Accordingly, the use of these two genera as biocontrol agents in the phyllosphere has been less studied than in the rhizosphere. However, a number of studies have already reported the effectiveness of *Pseudomonas* and *Bacillus* strains, isolated from the phyllosphere of different woody crops, namely lemon (Michavila et al., 2017) and apple (Mikiciński et al., 2016) trees, to control bacterial pathogens that infect their aboveground organs. Interestingly, isolates belonging to *Pseudomonas* (Zadeh et al., 2008; Krid et al., 2010) and *Bacillus* (Krid et al., 2010, 2012) were previously reported to be effective in antagonizing *Pss* in *in vitro* assays, as observed in our work. For the first time, *A. faecalis* isolates revealed the capacity to strongly inhibit *Pss in vitro* growth. This species has previously displayed ability to inhibit the growth of other phytopathogens under *in vitro* conditions (Lahlali et al., 2020), as well as capacity to induce plant defenses against fusarium wilt via lipopolysaccharide production (Patel et al., 2020). These features make this species as a potential candidate for OK biocontrol as well.

Most of the *in vitro* inhibition of *Pss* exhibited by tested antagonistic bacteria should have been caused by the induction of volatile organic compounds (VOCs) upon bacterial interaction. Indeed, eight bacterial strains out of the tested 15 strains able to antagonize *Pss*, increased their production of VOCs in co-culture. The antibacterial activity of produced VOCs by microbial antagonists has been previously reported and proposed as an effective biological control strategy against several phytopathogens (Ossowicki et al., 2017; Bui et al., 2019). These compounds may act as promoters of plant defense responses (Erb, 2018) and/or as inhibitors of phytopathogens growth (Enespa and Chandra, 2017). Furthermore, due to their highly diffusible capacity, microbial VOCs are considered ideal for biocontrol approaches because they do not require the contact between antagonist and pathogen to perform their activity (Contarino et al., 2019). Previous studies have shown VOCs potential from *Pseudomonas* (Agisha et al., 2019), *Bacillus* (Gao et al., 2018), and *Alcaligenes* (Gong et al., 2019) to inhibit the growth of different pathogens, such as fungal, oomycete, and nematode, as well as disease suppression caused by them. Besides VOCs, both siderophores and lytic enzymes could be also involved in the inhibition mechanism of *Pss* growth. An induction in the production of iron binding ligands (siderophores) was detected in three bacterial antagonists in the presence of *Pss*. Siderophores are known to sequester iron from the culture medium, making the iron unavailable to interacting bacteria (in this case *Pss*) and restricting their growth. Iron is essential for growth and pathogenesis of almost all species of phytopathogenic bacteria (Shanmugaiah et al., 2015; Pi and Helmann, 2017). Suppression of phytopathogens by bacterial antagonists through siderophore-mediated competition for iron has been already reported either in *in vitro* (Akter et al., 2016) or in field (Sasirekha and Srividya, 2016) conditions. There are some previous studies reporting the capacity to suppress several pathogens through siderophores production by *Pseudomonas*, *Bacillus* (review by Carmona-Hernandez et al., 2019) and *Alcaligenes* (Sayyed and Chincholkar, 2009) genera. From the tested bacterial antagonists, five revealed an induction on the lytic enzymatic activity (lipases or proteases) in the presence of *Pss*. Previous studies have similarly reported strong enhancement of these enzymes in bacterial antagonists during *in vitro* interaction with several plant pathogens (Amaresan et al., 2012; Geetha et al., 2014). We hypothesize that bacterial antagonists may inhibit *Pss* growth by secreting lipase and protease enzymes that will degrade pathogen cellular components. Indeed, it was within the bacterial isolates that displayed the greatest antagonistic activity toward *Pss* that the increment on the production of these enzymes was observed. This was particularly noticed for *A. faecalis* (D144; protease), *Pseudomonas oryzihabitans* (P271, lipase), and *B. amyloliquefaciens* (P41; lipase). Strains of these genera were previously reported to produced proteases and lipases that inhibit pathogen growth (Durairaja et al., 2018; Lahlali et al., 2020). The effective role of such enzymes as well as of siderophores in inhibiting *Pss* growth and germination should be confirmed. Accordingly, further research should test the effect of purified lipase, protease and siderophores from the antagonistic bacterial isolates on *Pss* growth and germination.

Among the different bacterial strains tested, the P41 isolate (previously identified as *B. amyloliquefaciens*) revealed the most promising antagonistic traits against *Pss* under *in vitro* conditions. This isolate obtained from twigs of cv. *Cobrançosa*, displayed the highest of all antimicrobial activities against *Pss*. Previous studies have similarly identified this species as one of the most efficient in inhibiting either bacterial (Abdallah et al., 2018) or fungal (Freitas et al., 2019) plant pathogens using *in vitro* conditions. In the present study, the mechanisms involved in *Pss* inhibition by *B. amyloliquefaciens* P41 probably comprise the secretion of inhibitory compounds/enzymes (VOCs and lipases) and competition for nutrients (through the production of siderophores). These mechanisms were also observed by other authors for this species (Kejela et al., 2016; Jamali et al., 2018). In addition, the genome analysis of *B. amyloliquefaciens* revealed the capacity of this species to produce other secondary metabolites aimed to suppress plant pathogens or enhance/mediate the defense responses of host plants against plant pathogens (Chowdhury et al., 2013). In agreement with *in vitro* assays, the inoculation of olive plantlets with *B. amyloliquefaciens* P41 together with *Pss* reduced significantly the OK disease severity, knots weight and *Pss* population size, when compared to pathogen (*Pss*) inoculated plants. Likewise, other studies have demonstrated the beneficial effects of *B. amyloliquefaciens* on disease suppression on other crops, such as tomato (Gautam et al., 2019), apple (Zhang et al., 2015), pistachio (Siahmoshteh et al., 2017), and lettuce (Chowdhury et al., 2013). Besides displaying biocontrol traits, several strains of *B. amyloliquefaciens* have been previously described as plant growth promoters (Asari et al., 2016; Abdallah et al., 2018). Contrasting with these reports, in the present study, *B. amyloliquefaciens* P41 did not promote plant growth when compared to non-inoculated control for most evaluated plant growth parameters. However, when *B. amyloliquefaciens* P41 was inoculated, either alone or in combination with *Pss*, a significant increase of both shoot dry weight and root water content were observed when compared to plants inoculated solely with *Pss*. This result suggests once more the increased capacity given to the host plant to deal with OK disease in the presence of *B. amyloliquefaciens* P41.

As far as we know, this study illustrates for the first time the potential role of *B. amyloliquefaciens* as a biological agent for controlling OK disease in olive trees, which has been an underexploited matter. Only *Bacillus subtilis* F1 (isolated from olive leaves) and *Bacillus mojavensis* A-BC-7 (isolated from the olive phylloplane) have demonstrated some efficiency, using *in planta* assays, in reducing the *Pss* population and knots size (Krid et al., 2012; Ghanney et al., 2016). Furthermore, the use of *B. amyloliquefaciens* as a biocontrol agent is of great significance because some strains of this species are already commercially available (e.g., Serenade, Bayer Crop Science; RhizoVital42, Abitep GmbH) for use as biocontrol agents against other plant pathogens (Chowdhury et al., 2015). However, we should emphasize that different strains could produce slightly distinct secondary products/enzymes with antibacterial activity, which would result in variable biocontrol efficiency. The comparison of *B. amyloliquefaciens* P41 and commercial available strains, regarding the biocontrol efficacy of produced antimicrobial compounds against OK disease would be an interesting approach for selecting the most appropriate BCA for controlling this olive disease.

## Conclusion

This work discloses the olive phyllosphere as harboring a number of bacterial strains with great potential to be used as biocontrol agents against *Pss*. In particular, *B. amyloliquefaciens* P41, revealed a great potential for the management of OK disease, by simultaneously promoting plant growth and reducing the disease severity of *Pss*-infected olive plantlets. Although promising results were obtained by using this strain in greenhouse assays, further experiments are needed to determine biocontrol effectiveness under field conditions and using different cultivars. The biocontrol mechanisms displayed by this strain also need to be further investigated.

## Data Availability Statement

The raw data supporting the conclusions of this article will be made available by the authors, without undue reservation.

## Author Contributions

DM performed the experiments, analyzed the data, and contributed to the manuscript writing. JP and TL-N contributed to the study design and manuscript editing. PB contributed to the study design, data analysis, and manuscript writing. All authors contributed to the article and approved the submitted version.

## Conflict of Interest

The authors declare that the research was conducted in the absence of any commercial or financial relationships that could be construed as a potential conflict of interest.
