# Supplementary material for: Screening the Olive Tree Phyllosphere: Search and Find Potential Antagonists Against Pseudomonas savastanoi pv. savastanoi
Source: Front Microbiol. 2020 Aug 25;11:2051. doi: 10.3389/fmicb.2020.02051 (PMC7477298; doi:10.3389/fmicb.2020.02051)
Supplement: Supplementary file 1 [file Table_1.docx]

Supplementary Material

# Supplementary Figures and Tables

## Supplementary Figures

**Supplementary Figure 1.** Development stages of olive knot disease on olive tree plants artificially inoculated with *Pseudomonas savastanoi* pv. *savastanoi* (scale: 1 = no knots; 2 = mild wound thickening; 3 = small knot at the wound base; 4 = small knots at both the base and top of the wound; 5 = knot completely covering the wound; 6 = knot larger than the wound).

## Supplementary Tables

**Supplementary Table S1**. Origin of bacterial strains assayed in this work. Epiphytic and endophytic bacteria were isolated and identified from distinct *Olea europaea* cultivars (*Cobrançosa* and *Verdeal Transmontana*) and different organs (leaves, healthy twigs and knots).

| **Cultivar** | **Organ** | **Epiphytes** | | **Endophytes** | |
| --- | --- | --- | --- | --- | --- |
|  |  | Isolate | Species | Isolate | Species |
| *Cobrançosa* | Leaves | P142 | *Sporosarcina aquimarina* | D77 | *Brevibacillus borstelensis* |
|  |  | P180 | *Kocuria rhizophila* | D87 | *Pseudomonas* sp. |
|  |  | P319 | *Bacillus velezensis* | D137 | *Curtobacterium* sp. |
|  |  | P381 | *Advenella* sp. | D164 | *Pseudomonas aeruginosa* |
|  |  | P401 | *Alcaligenes faecalis* | D282 | *Curtobacterium* sp. |
|  | Twigs | P40 | *Bacillus licheniformis* | D97 | *Erwinia toletana* |
|  |  | P41 | *Bacillus amyloliquefaciens* | D98 | *Alcaligenes faecalis* |
|  |  | P179 | *Brevibacterium frigoritolerans* | D127 | *Bacillus cereus* |
|  |  | P189 | *Microbacterium oxydans* | D287 | *Bacillus infantis* |
|  |  | P478 | *Bacillus plumilus* | D339 | *Solibacillus silvestris* |
|  | Knots | P141 | *Xanthomonas oryzae* | D63 | *Bacillus cereus* |
|  |  | P261 | *Paenibacillus* sp. | D96 | *Bacillus subtilis* |
|  |  | P276 | *Pseudomonas fragii* | D116 | *Bacillus altitudinis* |
|  |  | P31 | *Pseudomonas lutea* | D295 | *Bacillus safensis* |
|  |  | P174 | *Plantibacter flavus* | D296 | *Pseudomonas* sp. |
| *Verdeal Transmontana* | Leaves | P64 | *Bacillus amyloliquefaciens* | D29 | *Pseudomonas lutea* |
|  |  | P224 | *Pseudomonas* sp. | D75 | *Pseudomonas congelans* |
|  |  | P330 | *Alcaligenes faecalis* | D313 | *Pantoea vagans* |
|  |  | P362 | *Agrococcus versicolor* | D330 | *Alcaligenes faecalis* |
|  |  | P461 | *Pseudoclavibacter helvolus* | D333 | *Brevibacterium* sp. |
|  | Twigs | P181 | *Arthrobacter* sp. | D44 | *Alcaligenes faecalis* |
|  |  | P195 | *Curtobacterium herbarum* | D54 | *Paenochrobactrum* sp. |
|  |  | P226 | *Frondihabitans* sp. | D58 | *Pseudomonas aeruginosa* |
|  |  | P364 | *Serratia plymuthica* | D320 | *Alcaligenes faecalis* |
|  |  | P366 | *Brevundimonas* sp. | D329 | *Alcaligenes* sp. |
|  | Knots | P57 | *Pseudomonas corrugata* | D41 | *Bacillus cereus* |
|  |  | P271 | *Pseudomonas oryzihabitans* | D144 | *Alcaligenes faecalis* |
|  |  | P463 | *Pseudomonas* sp. | D303 | *Pseudomonas* sp. |
|  |  | P471 | *Serratia* sp. | D326 | *Alcaligenes* sp. |
|  |  | P433 | *Bacillus cereus* | D277 | *Bacillus subtilis* |

**Supplementary Table S2**. Growth inhibition (% growth rate reduction) of *Pseudomonas savastanoi* pv. *savastanoi* (*Pss*) and interacting bacterial isolates in dual culture, in relation to controls (single-cultures of *Pss* or interacting bacterial isolates). Datasets in bold represent the most promising isolates, displaying more than 50% of *Pss* growth inhibition while not being significantly inhibited by *Pss*, which were selected for evaluating the associated antagonism mechanisms. Statistical significance: *p <0.05; **p <0.01; ***p <0.001.

| **Epiphytes** | | |  | **Endophytes** | | |
| --- | --- | --- | --- | --- | --- | --- |
| **Isolate** | ***Pss*** | **Interacting isolate** |  | **Isolate** | ***Pss*** | **Interacting isolate** |
| P142 | 49.0±7.5** | 31.8±1.6 |  | D77 | 56.1±3.3*** | 48.7±4.1 |
| P180 | -34.4±22.5 | 29.9±28.5 |  | D87 | 11.7±8.0 | 57.7±3.9*** |
| P319 | -10.4±17.4 | -21.3±13.3 |  | **D137** | **78.2±6.5***** | **-108.1±17.5**** |
| P381 | 28.2±6.6 | 69.5±1.6** |  | D164 | 28.3±4.0 | -7.7±3.4 |
| P401 | 16.4±2.0 | 48.2±7.9* |  | **D282** | **50.2±13.4** | **14.4±2.5** |
| P40 | -20.1±14.1 | 55.1±22.0 |  | **D97** | **62.1±4.2***** | **-6.2±3.3** |
| **P41** | **85.2±0.8***** | **-150.6±24.2**** |  | **D98** | **69.2±2.1***** | **-25.4±10.2** |
| P179 | -0.6±3.0 | 49.5±3.8** |  | D127 | 52.5±2.8* | 71.3±6.9** |
| **P189** | **55.0±10.1**** | **2.7±11.4** |  | D287 | 20.2±19.5 | 65.5±3.5** |
| P478 | 29.8±7.9 | 42.9±6.3 |  | D339 | 24.8±3.4 | 3.5±4.8 |
| **P141** | **72.5±8.3***** | **19.7±4.6** |  | D63 | -59.1±18.2* | 71.1±12.0** |
| P261 | 58.8±6.4*** | 61.7±19.1* |  | D96 | 2.2±7.5 | -4.6±14.5 |
| P276 | 40.0±6.2* | 66.3±10.0* |  | D116 | 16.6±1.1 | 30.1±2.4 |
| P31 | 12.1±4.7 | 24.4±4.9 |  | D295 | 13.7±3.6 | 12.6±8.9 |
| P174 | 14.6±4.2 | 19.9±7.0 |  | D296 | 15.8±3.6** | -10.9±4.4 |
| P64 | 22.0±5.3 | 23.1±6.1 |  | D29 | 32.6±11.8 | 23.0±5.0 |
| **P224** | **54.6±3.3*** | **16.8±7.9** |  | D75 | 31.1±2.7** | 11.3±15.6 |
| P330 | 43.7±4.5*** | 4.4±14.5** |  | **D313** | **67.6±3.0***** | **-1.7±10.0** |
| P362 | 0.3±1.9 | 43.2±3.6 |  | D330 | 68.2±1.1*** | 45.6±1.1** |
| **P461** | **72.4±0.9***** | **-33.5±34.6** |  | D333 | -29.3±30.6 | 70.5±4.3 |
| P181 | 24.7±3.1* | 44.2±3.9 |  | D44 | 27.5±18.1 | 8.8±1.0** |
| P195 | 31.7±10.3 | 20.2±3.1 |  | **D54** | **50.8±5.3**** | **19.3±2.2** |
| P226 | 30.73±3.38* | 47.7±9.8 |  | D58 | 6.1±6.2 | 67.9±0.3* |
| P364 | 80.0±8.5** | 85.6±2.4* |  | D320 | -1.1±10.3 | 11.8±7.2 |
| P366 | 47.5±7.6 | 86.8±8.1** |  | D329 | 31.5±2.5 | -12.9±1.7 |
| P57 | 29.0±6.06 | 35.4±2.9 |  | D41 | 20.9±12.0 | 35.8±2.9 |
| **P271** | **68.70±10.8*** | **17.0±9.6** |  | **D144** | **75.4±0.3**** | **25.3±2.8** |
| P463 | 51.9±22.0 | 10.6±36.7 |  | **D303** | **52.4±2.2***** | **-14.6±10.3** |
| **P471** | **77.1±3.5**** | **38.8±13.5** |  | D326 | -8.8±12.6 | 54.1±3.7*** |
| P433 | 43.7±9.3* | 62.3±1.2*** |  | D277 | 12.9±2.4 | 34.2±9.1 |

**Supplementary Table S3**. Production of siderophores and lytic enzymes (proteases and lipases) by antagonistic bacterial isolates in co-culture with *Pseudomonas savastanoi* pv. *savastanoi* (*Pss*) or in single-culture (control). The values are the radius (mm) of orange (for siderophores) or clearing (for enzymes) halo formed around the antagonistic bacterial colony. Data are presented as means ± SD (n=5). Statistically significant differences (p<0.05) between co-culture and control, in each compound, are indicated by different letters.

| **Bacterial isolates** | **Siderophores** | | **Protease** | | **Lipase** | |
| --- | --- | --- | --- | --- | --- | --- |
|  | Co-culture with *Pss* | Control | Co-culture with *Pss* | Control | Co-culture with *Pss* | Control |
| P224 | 0.0±0.0a | 0.0±0.0a | 0.0±0.0a | 0.0±0.0a | 20.0±1.8a | 16.8±1.5a |
| P461 | 9.5±0.2a | 21.5±1.5b | 0.0±0.0a | 0.0±0.0a | 18.2±1.4a | 14.0±0.8a |
| P271 | 0.0±0.0a | 0.0±0.0a | 0.0±0.0a | 0.0±0.0a | 15.5±0.2a | 10.5±0.7b |
| P471 | 0.0±0.0a | 0.0±0.0a | 16.8±3.2a | 16.7±2.9a | 0.0±0.0 | 0.0±0.0 |
| P41 | 27.2±1.2a | 21.5±1.5b | 34.3±1.0a | 29.8±2.2a | 18.2±0.7a | 14.3±1.2b |
| P189 | 0.0±0.0a | 0.0±0.0a | 15.0±1.3a | 12.8±0.8a | 0.0±0.0 | 0.0±0.0 |
| P141 | 9.2±0.5a | 9.5±0.6a | 27.6±2.3a | 33.8±1.9b | 15.7±0.7a | 10.7±0.8b |
| D313 | 0.0±0.0a | 0.0±0.0a | 15.3±1.3a | 13.2±0.5a | 0.0±0.0 | 0.0±0.0 |
| D54 | 14.8±0.7a | 14.3±0.4a | 10.3±1.5a | 5.5±0.2b | 0.0±0.0 | 0.0±0.0 |
| D144 | 14.7±0.8a | 9.5±0.6b | 18.7±0.6a | 14.0±1.5b | 0.0±0.0 | 0.0±0.0 |
| D303 | 0.0±0.0a | 0.0±0.0a | 13.2±0.7a | 12.8±0.8a | 0.0±0.0 | 0.0±0.0 |
| D137 | 17.8±1.2a | 17.7±0.4a | 13.3±1.3a | 11.7±0.7a | 0.0±0.0 | 0.0±0.0 |
| D282 | 0.0±0.0a | 0.0±0.0a | 13.8±1.6a | 11.8±0.9a | 18.3±1.3a | 14.4±1.1a |
| D97 | 8.8±0.9a | 6.5±0.2b | 0.0±0.0a | 0.0±0.0a | 0.0±0.0 | 0.0±0.0 |
| D98 | 0.0±0.0a | 0.0±0.0a | 13.2±0.7a | 11.3±1.0a | 19.2±1.9a | 15.3±1.2a |

**Supplementary Table S4.** Contents of chlorophyll *a*, chlorophyll *b*, total chlorophyll and carotenoids on leaves of olive plantlets inoculated with the pathogen (*Pss*), with both antagonist and pathogen (*Pss* + P41), with the antagonist (P41) and with LB medium (negative control). Data are presented as means ± SE (n=5). Statistically significant (*p<*0.05) differences between treatments, in each day post-inoculation (DPI), are indicated by different letters.

| **Pigments** | **DPI** | | | | |
| --- | --- | --- | --- | --- | --- |
| Treatment | **14** | **28** | **42** | **56** | **70** |
| **Chlorophyll *a* (mg/g)** |  |  |  |  |  |
| Control | 1.09 ± 0.18^a^ | 1.32 ± 0.12^b^ | 1.40 ±0.07^b^ | 1.20 ± 0.17^a^ | 1.58 ± 0.20^a^ |
| *Pss* | 1.25 ±0.15^a^ | 1.16 ±0.18^b^ | 1.11 ±0.15^b^ | 1.69 ±0.16^a^ | 1.67 ±0.12^a^ |
| P41 | 0.90 ± 0.12^a^ | 1.06 ± 0.08^a^ | 0.92 ± 0.06^a^ | 1.27 ± 0.10^a^ | 1.21 ± 0.10^a^ |
| *Pss* + P41 | 1.29 ± 0.12^a^ | 1.32 ± 0.04^b^ | 1.54 ± 0.27^b^ | 1.68 ± 0.07^a^ | 1.63 ± 0.13^a^ |
|  |  |  |  |  |  |
| **Chlorophyll *b* (mg/g)** |  |  |  |  |  |
| Control | 0.48 ± 0.06^a^ | 0.50 ± 0.05^a^ | 0.55 ± 0.02^a^ | 0.50 ± 0.06^a^ | 0.67 ± 0.09^a^ |
| *Pss* | 0.52 ± 0.09^a^ | 0.46 ± 0.07^a^ | 0.45 ± 0.05^a^ | 0.70 ± 0.07^a^ | 0.74 ± 0.07^a^ |
| P41 | 0.35 ± 0.04^a^ | 0.44 ± 0.03^a^ | 0.39 ± 0.03^a^ | 0.55 ± 0.04^a^ | 0.53 ± 0.05^a^ |
| *Pss* + P41 | 0.51 ± 0.05^a^ | 0.54 ± 0.02^a^ | 0.62 ± 0.11^a^ | 0.71 ± 0.04^a^ | 0.69 ± 0.06^a^ |
|  |  |  |  |  |  |
| **Total chlorophyll (mg/g)** |  |  |  |  |  |
| Control | 1.57 ± 0.24^a^ | 1.82 ± 0.17^a^ | 1.94 ± 0.09^a^ | 1.70 ± 0.23^a^ | 2.26 ± 0.29^a^ |
| *Pss* | 1.78 ± 0.23^a^ | 1.62 ± 0.25^a^ | 1.56 ± 0.21^a^ | 2.39 ± 0.23^a^ | 2.41 ± 0.19^a^ |
| P41 | 1.26 ± 0.16^a^ | 1.50 ± 0.11^a^ | 1.31 ± 0.09^a^ | 1.82 ± 0.14^a^ | 1.74 ± 0.15^a^ |
| *Pss* + P41 | 1.80 ± 0.16^a^ | 1.86 ± 0.06^a^ | 2.16 ± 0.38^a^ | 2.39 ± 0.11^a^ | 2.32 ± 0.19^a^ |
|  |  |  |  |  |  |
| **Carotenoid (mg/g)** |  |  |  |  |  |
| Control | 0.20 ± 0.04^a^ | 0.27 ± 0.02^a^ | 0.27 ± 0.01^ab^ | 0.23 ± 0.03^a^ | 0.31 ± 0.04^a^ |
| *Pss* | 0.26 ± 0.03^a^ | 0.24 ± 0.03^a^ | 0.22 ± 0.03^ab^ | 0.33 ± 0.03^a^ | 0.32 ± 0.02^a^ |
| P41 | 0.21 ± 0.03^a^ | 0.23 ± 0.02^a^ | 0.19 ± 0.02^a^ | 0.25 ± 0.02^a^ | 0.24 ± 0.02^a^ |
| *Pss* + P41 | 0.27 ± 0.02^a^ | 0.26 ± 0.01^a^ | 0.31 ± 0.04^b^ | 0.32 ± 0.01^a^ | 0.30 ± 0.02^a^ |
